# Intraesophageal Migration of a Paraesophageal Hernia Mesh: A Case Report

**DOI:** 10.7759/cureus.24339

**Published:** 2022-04-21

**Authors:** Anass Idrissi, Omar Mouni, Mohamed Bouziane, Abdelaziz Fadil, Khalid Sair

**Affiliations:** 1 Visceral Surgery, Faculty of Medicine, Mohammed VI University of Health Sciences (UM6SS), Casablanca, MAR

**Keywords:** paraesophageal hernia, mesh repair, complication, recurrences, migration

## Abstract

Paraesophageal hernias (PEH) have a high recurrence rate, which can justify the use of a mesh during their repair. Mesh use in PEH repair is highly debated as it can lead to many complications like erosion and migration of the mesh, like in our case.

Here, we present a case of a 23-year-old woman operated on multiple occasions for a recurring PEH and who presented an intraesophageal migration of the mesh. Partial upper gastrectomy and lower esophagectomy were performed to remove the mesh and the recurrent hernia was repaired using primary sutures of the hiatus. The surgery was without complications and there are no signs of recurrence up to a year later.

Reoperation on a recurring PEH can be more difficult in case of mesh use in previous intervention and can lead to other complications like mesh erosion or migration, even so, some surgeons choose this option because it has a lower recurrence rate.

## Introduction

Paraesophageal hernias (PEH) have a high recurrence rate, which can justify the use of a mesh during their repair. However, the use of a mesh is not without risk, as it can lead to many complications including erosion and migration of the mesh [1].

Reoperating a recurrent PEH may not benefit the patient, that is why we must consider the severity of the symptoms and also consider other alternatives to simple repair such as esophageal lengthening and mesh use (even if it is controversial) [2].

We present here a case of a patient with a recurring PEH complicated by an intraesophageal mesh migration, a rare complication that made the redo surgery more difficult.

## Case presentation

We report a case of a 23-year-old patient who was admitted to Cheikh Khalifa Hospital, Casablanca, Morocco. The patient had a prior history of gastrointestinal reflux for which medication was given for about six months, but with no success in relieving the symptoms, an endoscopy was performed in which a PEH was discovered.

**Figure 1:**
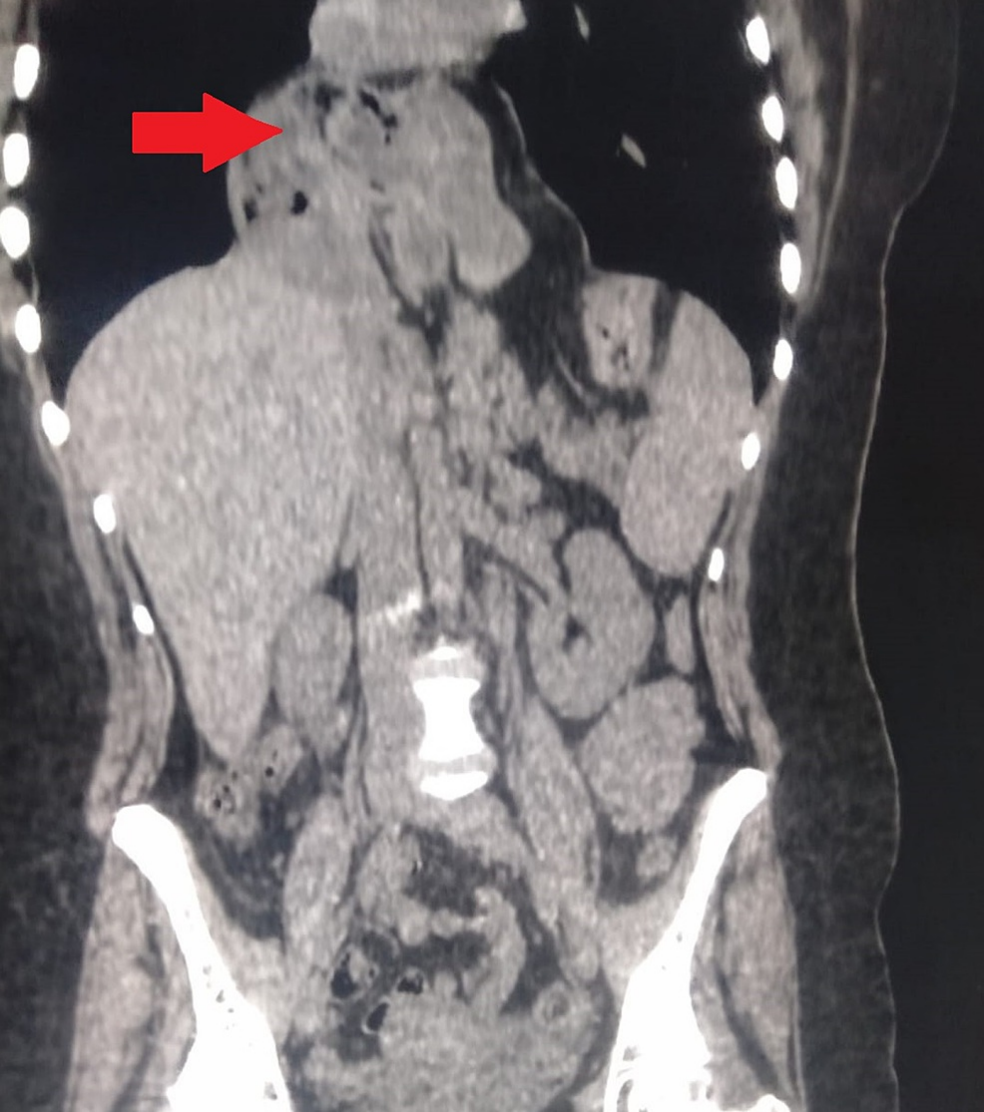
CT scan showing the paraesophageal hernia recurrence. Coronal scan showing the intrathoracic hernial sac (red arrow).

The patient had a first surgery, which consisted of a Nissen fundoplication and primary sutures of the hiatus for the hernia repair. Six months later, the patient suffered from severe dysphagia and an endoscopy found complete esophageal stenosis. A CT scan with IV contrast was done, which found an intrathoracic migration of the anti-reflux system. It is worth noting that the patient had no high-risk factors of recurrence (no chronic cough or constipation, no history of heavy weight lifting, and no history of smoking) (Figure 1).

**Figure 2:**
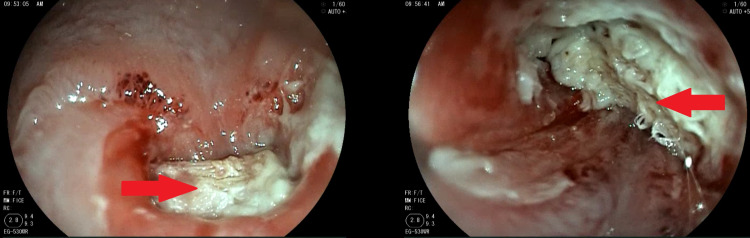
Endoscopic view showing intraluminal migration of the mesh. Intraesophageal localization of the mesh (red arrows).

A redo surgery was done, which consisted of unwrapping of the Nissen fundoplication and a hernia repair. After complete resection of the hernia sac, reinforced by a non-absorbable double-sided mesh fixed on the diaphragm and surrounding the esophagus, no anti-reflux procedure was done, and we decided the placement of a gastrostomy or a jejunostomy was not necessary, as the patient went back to normal feeding habit after the surgery (liquid for the first day, then normal the following days). The immediate follow-up was without complication. No swallow-up studies were performed, as they were unavailable. The patient was readmitted to the emergency department a month later with acute appendicitis for which a laparoscopic appendectomy was performed without any complication. A month after that, the patient was readmitted with gradual dysphagia becoming severe before admission, for both liquids and solids. An endoscopy was performed, which showed an intraesophageal migration of the mesh 33 cm from the incisors (Figure 2).

**Figure 3:**
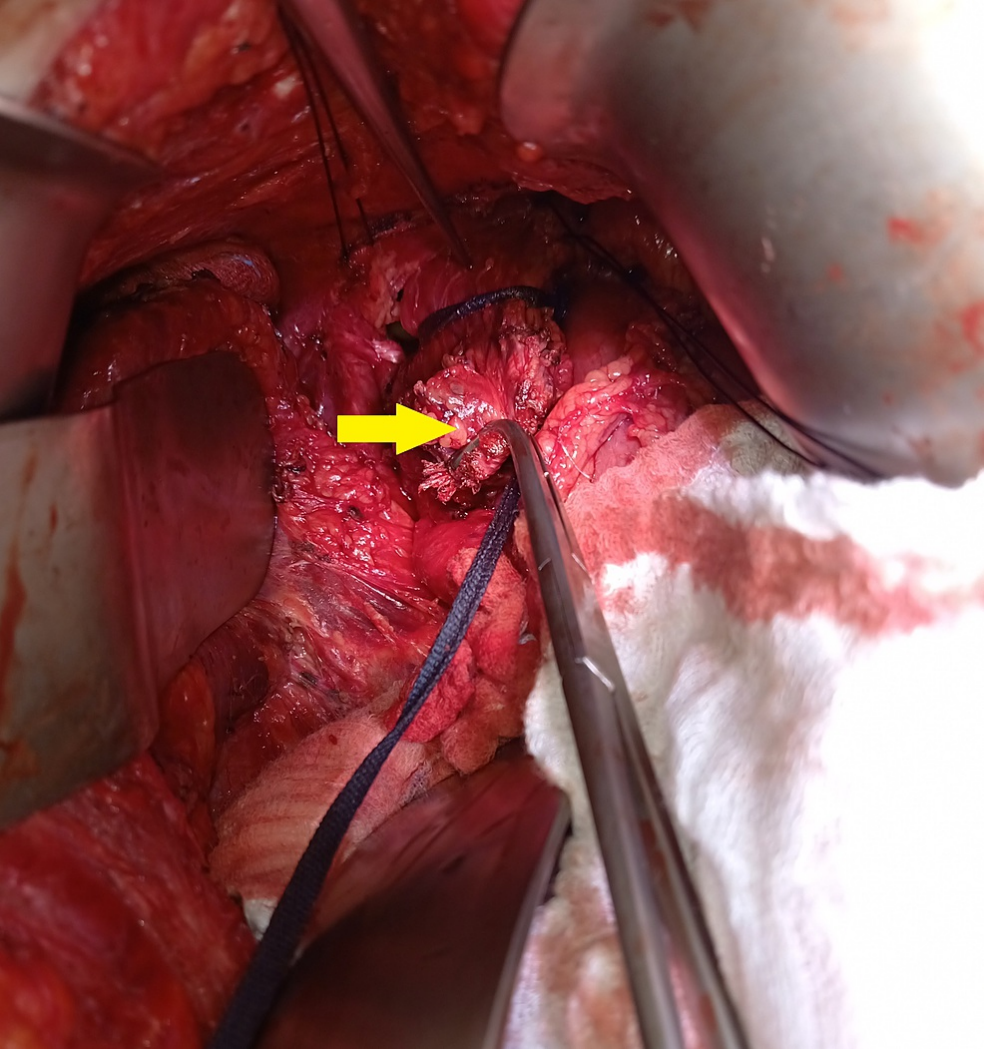
Perioperative view showing mesh adhering to the esophagus. Mesh adhering to the esophagus (yellow arrow).

**Figure 4:**
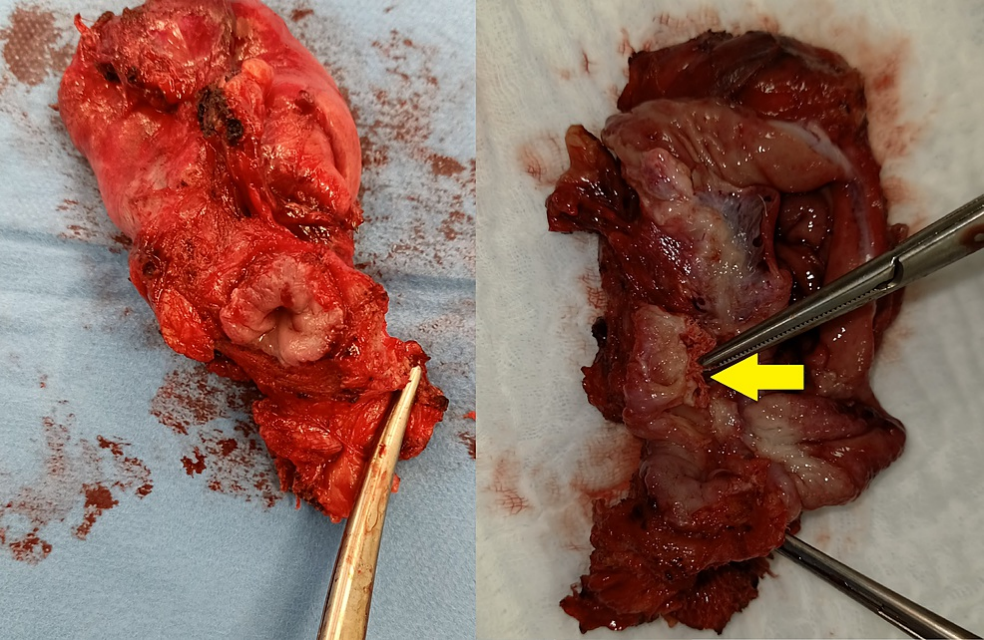
Operative specimen showing intraesophageal migration of the mesh. Mesh located inside the esophageal lumen (yellow arrow).

The patient was re-operated for the fourth time. After an open laparotomy, a distal esophagostomy with a partial upper gastrectomy was performed after opening the esophageal hiatus. Only a small upper section of the stomach was removed using a universal stapler, and the esophagus was sectioned above the mesh, as it was completely adherent and could not be removed on its own (Figures 3, 4). The continuity of the digestive tract was restored immediately by a manual gastroesophageal anastomosis and the esophageal hiatus closure was done with primary sutures with a non-absorbable thread. It is worth noting that the surgery was performed through the abdomen.

The immediate follow-up was without complications. The patient was discharged on day four. A year later, a control endoscopy was done with no signs of recurrence.

## Discussion

The high recurrence rate of PEH can prompt some surgeons to consider the use of mesh in their repair. The benefits of mesh augmentation are debated in light of its risks [1], as our case illustrates here. Even when we use a mesh to treat a recurrent PEH, it can lead to other complications (mesh migration), and even if they are rare, they can be difficult to treat and require a partial resection of the stomach and the esophagus, as seen in our case.

The type of mesh used is primarily based on personal experience and results from randomized trials and observational studies [3]. An analysis of the NSIQP (National Surgical Quality Improvement Program) of the American College of Surgeons [3] revealed 40% use of mesh yearly and there was no association with a higher incidence of postoperative complications. A survey among the Society of American Gastrointestinal and Endoscopic Surgeons (SAGES) members [4] showed that 25% of respondents use a mesh (biomaterial (28%), polytetrafluoroethylene (25%), and polypropylene (21%)) regularly (more than half of PEH repair). The recurrence rate was still high: 54% vs. 59% for primary repair (p = 0.7). There is variable evidence supporting mesh use [4-8], but even so, its use remains high [9-11] because of its long-term recurrence rate [12].

In our case, we opted for the use of a composite mesh with a collagen film on one side to minimize the risk of erosion. This was revealed to be ineffective. We can only theorize that during appendicitis, the patient presented may have a bacterial translocation that could have exacerbated the inflammatory response around the mesh. This may have led to a more pronounced erosion that led to an intraesophageal migration of the mesh.

When considering the type of mesh, synthetic meshes like polypropylene and polytetrafluoroethylene (PTFE)-based mesh can lead to more frequent erosions [2], but this can be explained by the fact that synthetic meshes are simply more frequently used as argued by Antoniou et al. [7]. In our case, we opted for synthetic mesh because the biological mesh is simply not available in our country.

The use of a mesh may lead to a reduced long-term recurrence, according to some studies [7,13], but it can lead to a more difficult redo surgery, as in our case, for which we had to perform a surgical resection to remove the mesh completely. Other more recent studies suggest that mesh augmentation might be associated with less short-term recurrences, and the biological mesh was associated with improved short-term quality of life. However, these advantages were offset by more dysphagia [11], which is why most experts recommend mesh use only in specific circumstances [14]. Finally, robotic surgery may eventually make it easier, compared to the laparoscopic approach, when reoperating a patient like ours, considering the higher dexterity it offers [15], but its practical use remains limited because of its cost, especially in a country like ours.

## Conclusions

Mesh repair in PEH may have a lower recurrence rate and may lead to a lower rate of redo surgeries, but the complications that occur may lead to more difficult redo surgery. Our case, even though it is rare, illustrates this, as we had to perform a partial gastrectomy and esophagectomy to remove the mesh that migrated inside the digestive system.

The mesh type may play a role in the complication rate, with synthetic mesh being more implicated. The presence of an intraperitoneal infection such as appendicitis may also play a role, although that remains to be proven.
